# A modified version of Moran's *I*

**DOI:** 10.1186/1476-072X-9-33

**Published:** 2010-06-29

**Authors:** Monica C Jackson, Lan Huang, Qian Xie, Ram C Tiwari

**Affiliations:** 1 Department of Mathematics and Statistics, 4400 Massachusetts Ave NW, American University, Washington, DC 20016, USA; 2 Office of Biostatistics, Center for Drug Evaluation and Research, Food and Drug Administration (FDA), 10903 New Hampshire Avenue, MD 20993-0002, USA

## Abstract

**Background:**

Investigation of global clustering patterns across regions is very important in spatial data analysis. Moran's *I *is a widely used spatial statistic for detecting global spatial patterns such as an east-west trend or an unusually large cluster. Here, we intend to improve Moran's *I *for evaluating global clustering patterns by including the weight function in the variance, introducing a population density (PD) weight function in the statistics, and conducting Monte Carlo simulation for testing. We compare our modified Moran's *I *with Oden's I*_pop _for simulated data with homogeneous populations. The proposed method is applied to a census tract data set.

**Methods:**

We present a modified version of Moran's *I *which includes information about the strength of the neighboring association when estimating the variance for the statistic. We provide a power analysis on Moran's *I*, a modified version of Moran's *I*, and I*_pop _in a simulation study. Data were simulated under two common spatial correlation scenarios of local and global clustering.

**Results:**

For simulated data with a large cluster pattern, the modified Moran's *I *has the highest power (43.4%) compared to Moran's *I *(39.9%) and I*_pop _(12.4%) when the adjacent weight function is used with 5%, 10%, 15%, 20%, or 30% of the total population as the geographic range for the cluster.

For two global clustering patterns, the modified Moran's *I *(power > 25.3%) performed better than both Moran's *I *(> 24.6%) and I*_pop _(> 7.9%) with the adjacent weight function. With the population density weight function, all methods performed equally well.

In the real data example, all statistics indicate the existence of a global clustering pattern in a leukemia data set. The modified Moran's *I *has the lowest p-value (.0014) followed by Moran's *I *(.0156) and I*_pop _(.011).

**Conclusions:**

Our power analysis and simulation study show that the modified Moran's *I *achieved higher power than Moran's *I *and I*_pop _for evaluating global and local clustering patterns on geographic data with homogeneous populations. The inclusion of the PD weight function which in turn redefines the neighbors seems to have a large impact on the power of detecting global clustering patterns. Our methods to improve the original version of Moran's *I *for homogeneous populations can also be extended to some alternative versions of Moran's *I *methods developed for heterogeneous populations.

## Background

Global indices of spatial autocorrelation have been used to evaluate the degree to which similar observations tend to occur near each other [1-4]. Spatial autocorrelation among disease counts or incidence proportions may reflect real association between cases due to infection, or perceived association based on a spatial aggregation of similar values. Moran's *I *[5] is a widely used global index that measures the similarity for values in neighboring places from an overall mean value and reflects a spatially weighted form of Pearson's correlation coefficient. The traditional calculation of Moran's *I *for disease cases does not account for population heterogeneity, so that, its application to disease rates or proportions may result in indication of spatial correlation that is completely due to the spatial proximity of population sizes, but not due to the similarity among the disease rates. Several alternative versions of Moran's I have been proposed to account for heterogeneous populations, for example by Oden [6], Waldhor [7], Walter [2], Assuncao and Reis [8], and Waller et al. [9].

In this article, we intend to improve the original version of Moran's *I *[5] that tests the similarity of numbers (e.g., cases) in neighboring geographic units, by doing the following: incorporating a weight function in the variance computation, introducing the population density (PD) weight function, and conducting Monte Carlo simulation for testing global clustering pattern. The weight function is not only included in the differences of the geographic unit's cases (e.g., county cases) from the overall mean, but also in the calculation of the variance. We also expand the definition of neighbors to a broader concept in the construction of Moran's *I *(e.g., all geographic units included in a pre-specified geographic range will be considered to be neighbors of the geographic unit in the center). Statistical inference is conducted assuming a null hypothesis of constant risk instead of a normal distribution for the statistic. The proposed approaches for improving the original Moran's *I *can also be applied to some alternative Moran's *I *methods developed for heterogeneous population data, such as the rate version of Moran's *I *and the normalized Moran's *I *[10].

A simulation study is performed to evaluate the power of both Moran's *I *and modified Moran's *I *for data generated with varying local and global clustering patterns. The statistic I*_pop _is also selected to be evaluated in the simulation study because I*_pop _was developed specifically to improve the original Moran's *I *for heterogeneous population data, and I*_pop _achieved better power than Moran's *I *as discussed in Jackson et al. [11]. We also compared these statistics with a traditional weight function definition that assigns a 0/1 to each neighboring location, representing a non-neighbor/neighbor and expanding the weight function definition that includes more information such as the population density of the surrounding neighbors [3].

The outline of this article is as follows: We describe the original Moran's *I *and construct the Modified Moran's *I*. We also describe Oden's I*_pop_. An extensive simulation study was carried out to compare the power of these statistics in identifying the global spatial heterogeneity. We illustrate the application of the methods using leukemia incidence data at the census tract-level from upstate New York. Results and a discussion conclude this article.

## Methods

### Observations and Locations

Define *y*_*i *_as the number of cases and *n*_*i *_as the population at risk at geographic unit *i *where *i *= 1, ..., *N *with *N *being the total number of geographic units (e.g. census tracts or counties). Let *w*_*ij *_be the weight assigned to the pair of geographic units *i *and *j *(*i *≠ *j*), which reflects the strength of the relationship between geographic units *i *and *j*.

### Moran's *I*

Moran's *I *[5] is defined as(1)

where , . We note that *y*_*i *_are counts, however, alternative versions of Moran's *I *use continuous values. The weight in equation (1) is commonly defined based on adjacent neighbors (Adj) and is written as(2)

The weight *w*_*ij *_in Moran's *I *and its extensions are usually defined as in equation (2) (neighbor matrix). However, the weight function, *w*_*ij*_, can be defined in many other ways (see Song and Kulldorff [12] and Griffith [13]).

Let

Then, we can rewrite Moran's *I *as(3)

The value of *I *usually ranges between -1 and 1 and the expected value is . However, the range of *I *depends on the values of the weight function [3]. Positive values of *I *are associated with strong geographic patterns of spatial clustering, negative values of *I *are associated with a regular pattern, and a value close to zero represents complete spatial randomness. Note that areas with different population sizes were given the same weight (0 or 1) in Moran's *I*. The measure *y*_*i *_is the geographic unit's count, which does not include the geographic unit's population information. If a dataset has spatial correlations or clustering patterns due to the heterogeneous population sizes, the original Moran's *I *using counts will identify the clustering pattern which may be due to the spatial similarity of the population and not the spatial clustering pattern as desired.

### Modified Moran's *I*

In the definition of Moran's *I *(3), we notice that the weight is included in the numerator *s*_*yy,w*_, but not in the denominator . However, the "neighbors" who contribute more to the differences, , should also contribute more to their variation. Therefore, we proposed a Modified Moran's *I*, which is defined as(4)

where  in (1) is modified to depend on the weights *w*_*ij *_as

Thus, when *w*_*ij *_are non-zero constants (homogeneous weights),  and *I*_*w *_= *I*, since

The weights in  are taken to depend on the observations *y*_*i *_and *y*_*j*_, (inhomogeneous weights). Substituting  in (4), the Modified Moran's *I *is expressed as

Note that . Therefore, *I*_*w *_≤ *I *if the term, . Since *I*_*w *_depends on the choice of the weight function, its mathematical properties such as moments and asymptotic distribution can possibly be obtained using the functional central limit theorem for sums of weighted random variables; however, we have not explored these issues here. The range of *I*_*w *_may be outside the interval [-1,1]. The expected value of the modified Moran's *I *depends on the weight function and the distribution of *I*_*w *_is not tractable, therefore, we use Monte Carlo simulation procedure to obtain the empirical distribution for statistical inferences.

We consider here another common weight function called, the *population density adjusted exponential weight *(PD) [12], that allows for sparsely populated areas to include a larger geographic region to be incorporated in the weights than densely populated areas. Then, the scale of the spatial clustering can be adjusted based on the population density. This weight function is given by(5)

where *d*_*ij *_is the distance between geographic units *i *and *j*,  and *m*_*i *_= max{*j*:*u*_*j*(*i*) _≤ *λ*}. The population density across geographic unit *i *and its *j *nearest neighbors is defined as *u*_*j*(*i*)_. In this case, *u*_*j*(*i*) _represents the total population count of the larger region comprised of geographic unit *i *and all of its neighbors *j*. The parameter *λ *is chosen by the user and allows the user to view the population density as a measure of spatial clustering, where large (small) *λ *is more sensitive to larger (smaller) clustering patterns. We define the parameter *λ *to be 50% of total population in this study, where we wanted a significant amount of neighbors included in the analysis. Note that *k*_*i *_will be larger in areas that are sparsely populated (allowing for greater distances to be incorporated in the weight function) and smaller in areas that are densely populated (allowing smaller distances to be incorporated in the weight function). Therefore, the population density of the geographic region is incorporated in this weight function.

Note that since both *S*_*yy,w *_and *S*_*y,w *_depend on the weights, *W*_*ij*_, *I*_*w *_is more sensitive to the variability in the weights than *I*. And, there is more variability in the weights Adj in (2) as compared to the weights PD in (5).

### Oden's I*_pop_

As discussed in the introduction, I*_po__*p *_was developed as an alternative or Moran's *I *specifically for data with heterogeneous population. As an alternative version of Moran's *I*, Oden [6] derived the statistics I*_po__*p *_to test for global spatial autocorrelation adjusting for population heterogeneity. His statistic is defined as(6)

where , *v*_*i *_= *n*_*i*_/*n*_+_, *v*_*j *_= *n*_*j*_/*n*_+_, *e*_*i *_= *y*_*i*_/*y*_+_, *e*_*j *_= *y*_*j*_/*y*_+_, and . Oden noted that symmetry is not required for I*_pop _and *w*_*ii *_≠ 0 (but can be fixed at any specified value). In order to capture the variability present in a region, Oden includes the first term in the numerator which is used to model the spatial variation in a manner similar to the conventional chi-squared test for heterogeneity of rates.

### Power study

To compare the performance of modified Moran's *I *with Moran's *I*, we performed a power analysis using a simulation study. The *y*_*i *_is defined as the number of cases in our study; however, it can also be taken as rates or normalized cases. We used a homogenous population of *n*_*i *_= 5,000 for *i *= 1 to 3,109 for each of the 3,109 regions that represented the counties of the U.S. (resulting in a total population of *n*_+ _= 15,545,000.) We simulated regional count data sets under the null hypothesis of constant relative risk(7)

where (*y*_1_, *y*_2_, ..., *y*_3109_) are regional counts generated from the multinomial distribution. The total number of cases *y*_*+ *_here is always the same as the total number of cases in the corresponding data simulated under the alternative hypothesis.

We simulated regional count data sets under the alternative hypothesis(8)

where , *r*_*i *_is the relative risk at geographic unit *i*, which is not constant under the alternative hypothesis. The various value used for *r*_*i *_are based on the type of spatial pattern simulated as described in the following sections.

### Local cluster pattern

**Figure 1:**
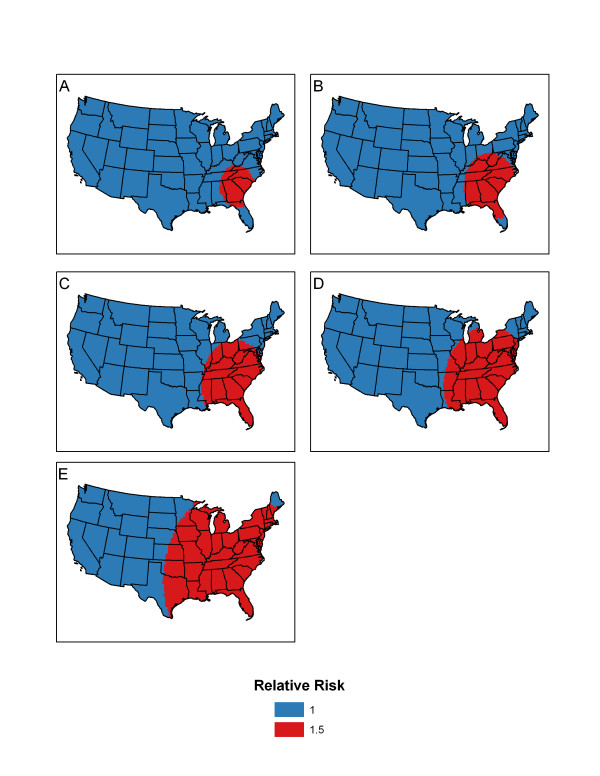
**Cluster pattern**. Simulated spatial clusters representing 5% (A), 10% (B), 15% (C), 20% (D), and 30% (E) of the total population.

First we selected Columbia County located in GA as the center of the cluster. Then using homogenous populations of 5,000 for each of the 3,109 counties of the U.S. (resulting in a total population of 15,545,000), we created a cluster that contained 5% of the total population centered at Columbia County. The relative risk for this cluster was 1.5 (with all other counties not included in the cluster having a relative risk of 1.) We repeated these steps to create clusters that contained 10%, 15%, 20% and 30% of the total population. Figure 1 shows the simulated clusters.

Our power study is based on homogeneous populations in order to reduce any confounding effects that occur when using heterogeneous populations with varying relative risk. Waller et al. [9] state there is an impact of local geography (in particular, population density) on power comparisons between statistical tests of spatial pattern. Therefore we study homogenous populations in the simulation study to remove the effect of the population.

### Global spiral clustering pattern

**Figure 2:**
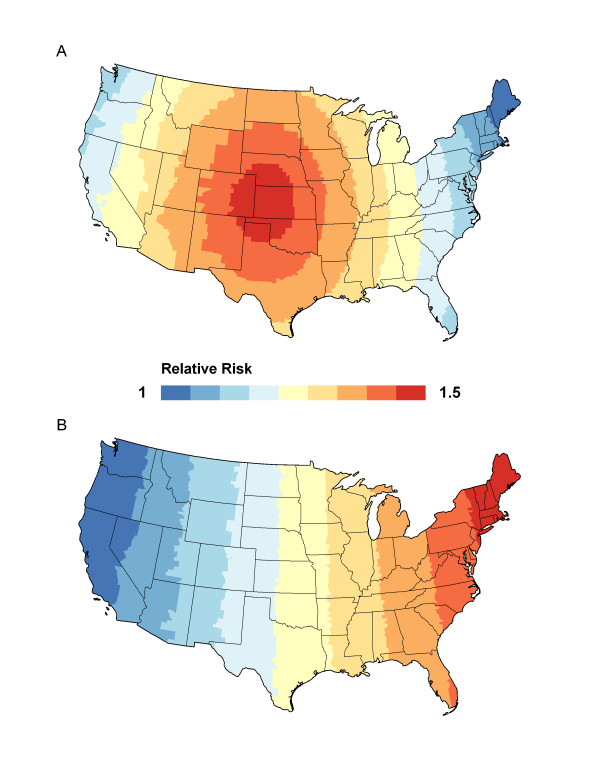
**Global pattern**. Simulated global spiral (A) and linear (B) spatial clustering patterns.

To simulate data that represent a large global clustering pattern, we used the following method. First we located a county in the center of the U.S. (Scott County KS). Then we included as the central cluster a set of counties (with Scott County as the center) that included 2% of the total population (recall that each county has a homogenous population of 5,000.) The relative risk for this central cluster is 1.5. Then, we decreased the relative risk gradually around the central cluster until the smallest risk is 1. The risk was gradually decreased in 100 equal intervals from 1.5 to 1. Figure 2A shows the simulated pattern divided into 9 groups for a simplified visualization.

### Global linear clustering pattern

The final pattern simulated is a west-east trend with a linear increasing function. We simulated a minimum relative risk of 1 on the west coast and a maximum relative risk of 1.5 on the east coast. The monotone increasing function is defined as(9)

where *a *is an integer from 0-99. The longitudinal coordinates of the continental United States ranges from -124.161 to -67.623. We divided this large interval into 100 sub-intervals of equal length where each interval corresponds to a value of *a*. Figure 2B shows the simulated data set.

### Calculating power

We used Monte Carlo simulation methods to assess the power of rejecting the null hypothesis by simulating 10,000 data sets using the multinomial distribution defined in equation (7) under the hypothesis of equal relative risk. The statistics (Moran's *I*, Modified Moran's *I*, and I*_pop_) obtained from the simulated null data are used to construct the empirical distribution of the statistics. The 95^th ^percentile for each of the statistics is defined as the critical value.

Then, Moran's *I*, Modified Moran's *I*, and I*_pop _were calculated for each of the 1000 data sets simulated under the alternative hypothesis using the multinomial distribution defined in equation (8) with the clustering pattern shown in Figures 1 and 2. Power is calculated as the percentage of values out of the 1000 replicated data sets that exceed the critical point obtained from data under the null hypothesis.

## Results

Our power analysis and simulation study shows that the Modified Moran's *I *achieved higher power than Moran's *I *and I*_pop _for evaluating the global and local clustering patterns on geographic data with homogeneous populations. All results are provided in Table 1.

**Table 1:** Powers (%) for Modified Moran's *I*, Moran's *I*, and I*_pop _for local and global spatial patterns with population density (PD) and adjacent (Adj) weight function.

		Modified Moran's *I*	Modified Moran's *I*	Moran's *I*	Moran's *I*	I^*^_pop_	I^*^_pop_
		Adj	PD	Adj	PD	Adj	PD
Percent of population in cluster							
						
**Local**	5	43.4	67.3	39.9	67.1	12.4	59.7
	10	80.3	99.3	76.7	99.4	27.8	99.2
	15	96.7	100	94.9	100	46.5	100
	20	99.2	100	98.6	100	61.8	100
	30	99.9	100	99.8	100	74.5	100

**Global**	Spiral	25.3	99.9	24.6	99.9	7.9	99.6
	Linear	27.0	99.6	24.3	99.6	7.4	99.0

### Results for the local cluster pattern

We experimented with cluster patterns that contained between 5% to 30% of the total population. We found that the larger the percent of the population within the cluster, the greater the power for all methods. Modified Moran's *I *has a higher power for all local cluster patterns. When the Adj weight function is used with 5% of the total population, we find that Modified Moran's *I *has a power of 43.4%, which is higher than the power for both the Moran's *I *(39.9%) and I*_pop _(12.4%). The same is true when for patterns with single cluster including 10%, 15%, 20%, and 30% of the total population. When the cluster reached 30% of the population, we obtained the highest power of 99.9% for Modified Moran's *I*, 99.8% for the Moran's *I *and 74.5% for I*_pop_.

When the PD weight function is used for cluster with 5% of the population we obtained powers of 67.3, 67.1, and 59.7% for Modified Moran's *I*, Moran's *I *and I*_pop_, respectively. Modified Moran's *I *performed slightly better in this scenario and similar for cluster with 10% of the population. When clusters with 15%, 20%, and 30% of the total population were used, all methods with the PD weight function performed equally with a power of 100% since the spatial pattern is very strong.

The statistics with the PD weight function obtained higher powers (compared to the Adj weight function) in all cases in our simulation study. Recall that the PD weight function incorporates a larger number of spatial neighbors compared to the Adj weight function; therefore, it includes more information for global clustering evaluation and has better powers for global spatial patterns. In general, there is no "best" definition of weights and weights can be based on distances or on other influences (e.g., how far a location is away from a contaminated water source)[14]. For a spatial analysis, weight functions are often chosen to have a spatial scale equivalent in size to the hypothesized cluster [15].

### Results for the global spiral clustering pattern

For the global clustering pattern (as shown in Figure 2A), Modified Moran's *I *(power = 25.3%) performed better than both Moran's *I *(24.6%) and I*_pop _(7.9%) with the Adj weight function. With the PD weight function, all methods performed equally well with a power very close to 100%.

### Results for the global monotone clustering pattern

Finally for the monotone pattern simulated as shown in Figure 2B, Modified Moran's *I *yielded the highest power of 27.0% compared to 24.3% for Moran's *I *and 7.4% for I*_pop _for the Adj weight function. For the PD weight function, Modified Moran's *I *had a power of 99.6% which is equal to the power of Moran's *I *and I*_pop_.

### Application

**Figure 3:**
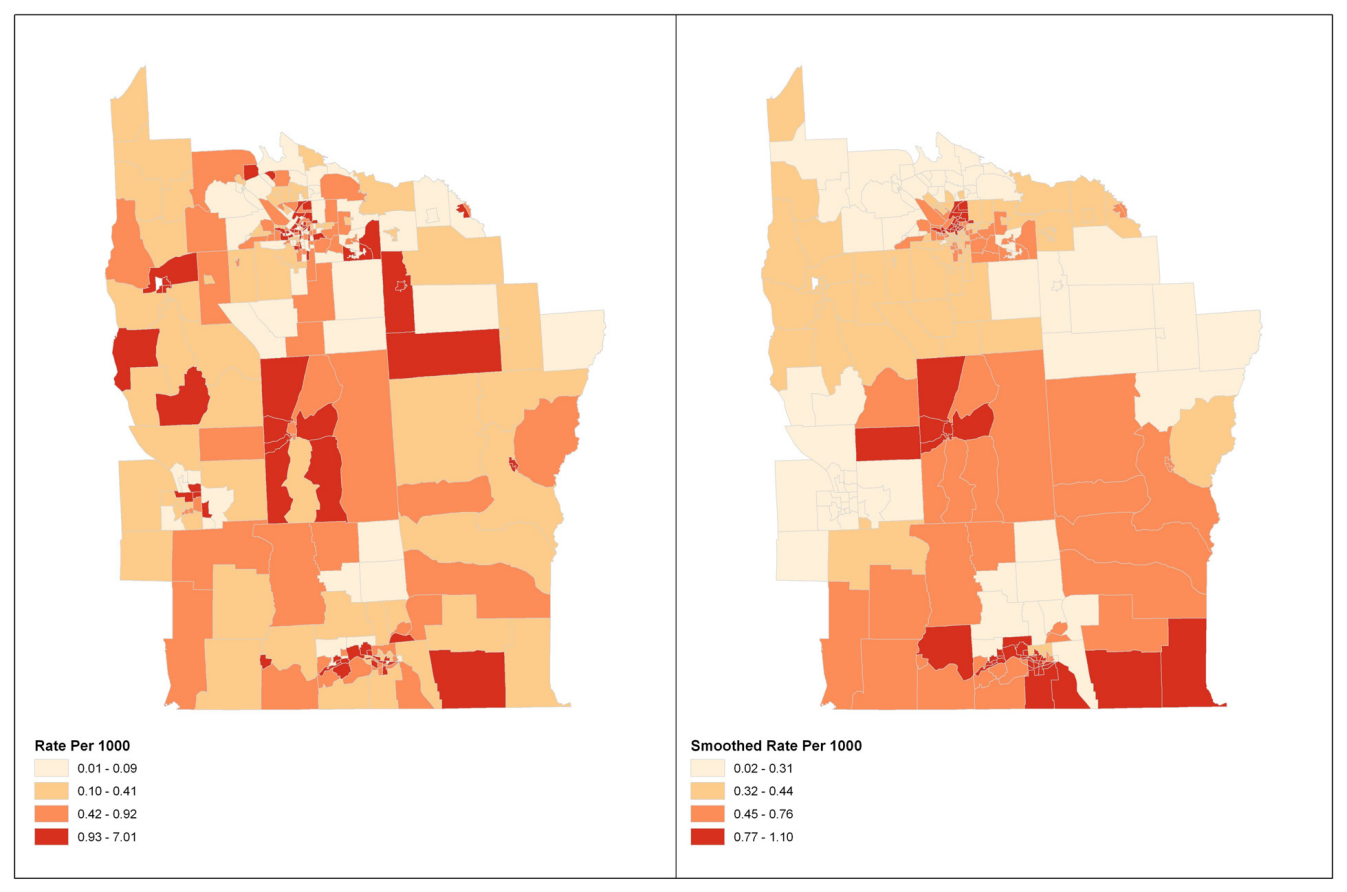
**Application**. Raw (left) and smoothed (right) rates of leukemia by census tract in eight counties of upstate New York from 1978-1982.

For illustration, we consider the New York leukemia data set introduced by Turnbull et al. [16] and presented in Waller and Gotway [3] and Jackson and Waller [4]. The data include the number of cases of leukemia and the population at risk for a region consisting of eight counties (Broome, Cayuga, Chenango, Cortland, Madison, Onondaga, Tioga, and Tompkins) in upstate New York for the years 1978-1982. The data are collected at the census tract-level and are presented as rates at the census tract-level in Figure 3 (with raw rates on the left and smoothed rates using the 10 nearest neighbors on the right) and include a total of 592 reported cases of leukemia among 1,057,673 people at risk. The mean population per census tract is 1,339 with a standard deviation of 1,144. It is difficult to observe any global clustering trend in the raw rate map. Therefore we generated a smoothed map (Figure 3 right) using headbang [17] with the nearest neighbor parameter set at 10, which basically maps the average of the rates over the 10 nearest neighbors instead of the original rate for a particular tract. The smoothed map reveals a possible global trend with lower rates in the northern part of the region and higher rates in the southern part of the 8 county regions.

Using the New York leukemia data set, we obtained p-values for Moran's *I*, Modified Moran's *I *and I*_pop _with the PD weight function. We find that Modified Moran's *I *has the lowest p-value (.0014) followed by Moran's *I *(.0156) and I*_pop _(.011). All three methods detected a global trend in this data (with significance level of *α *= 0.05), however, Modified Moran's *I *had the most significant p-value. This result is consistent with the finding from Waller and Gotway [3] that there exist global clustering pattern in the Leukemia rates in upstate New York.

## Discussion and Conclusion

In conclusion, we improved the original Moran's *I*, and conducted a simulation study to evaluate the performance of the proposed method. We considered various simulated regional patterns in data that involved local cluster patterns and global clustering patterns. The five local cluster patterns used formed a single cluster in the eastern part of the U.S with either 5%, 10%, 15%, 20%, or 30% of the population included in the cluster with a relative risk to all other regions of 1.5. The two global patterns involved simulating a west to east linear trend, and a pattern resembling a cluster in the center with 2% of population with the spatial correlation slowly decreasing until you reach the east and west coast with a relative risk of 1.5. We also applied the proposed method to a census tract dataset, which has a more homogeneous population than state or county level data (however, census tract data still has heterogeneous population [18].) The proposed approach for improving the original Moran's *I *(for homogeneous population data) can be applied to the rate version and the normalized version of Moran's *I*, which may be more suitable for analyzing data with heterogeneous populations. Future research may be conducted to explore the performance of those methods for data with heterogeneous populations. Similar idea can also be applied to local indicators of spatial association (LISA) [19] for cluster detection in future work.

Modified Moran's *I *with the adjacent weight function (Adj) achieved higher power for the simulated local and global cluster patterns than Moran's *I*, and the modified Moran's I with PD has similar performance compared with Moran's *I*. We compared the modified Moran's *I *with I*_pop _as well, since the latter I*_pop _was developed to be an alternative of the original Moran's *I *for data with heterogeneous populations. However, I*_pop _does not always perform well on homogeneously populated data as shown in our simulation study.

For the local clustering patterns, the power for Modified Moran's *I *increased as the percentage of the population included in the cluster (cluster size) increased for both weight functions. For the global clustering patterns, modified Moran's *I *achieved higher power than both of the other statistics. The power for the global patterns are lower than for the local cluster patterns, since the spatial size of the area with the largest relative risk (1.5) is much larger in the local cluster patterns compared to the global patterns. There are other issues that affect power of identifying a global clustering pattern. For example, Lindsey [20] states that there are problems with building models based on nearest neighbors due to edge effects. We evaluate the effect of the cluster size in the simulation study, but not the edge effect. A cluster on the edge may lead to a lower power of identifying the clustering pattern.

Note that there is no inflated type 1 error since the proposed test is for a global clustering pattern evaluation (not for cluster detection) and Monte Carlo procedure is used for statistical inference. The type I error, which reflects the chance of the method identifying a spatial pattern when there is none, is controlled at the alpha level (0.05). Also, when there is a spatial pattern, the proposed Moran's *I *can identify the pattern with reasonable power as shown in the simulation study.

It turns out that the weight function played a large role in the method performance. The adjacent weight function only has two values (0 for a non-adjacent-neighbor and 1 for an adjacent neighbor). If the weight is 0 for a pair geographic units *i *and *j*, the difference between the geographic unit *i *and *j *is not evaluated in the formulation of the statistics (e.g. Moran's *I *and *I*_*pop*_). Since the number of adjacent neighbors for all the counties in the continental U.S. ranges from 0-14 (see Jackson et al. 2009), there is only a limited number of pairs of geographic units that are evaluated in the statistics when the Adj weight function is used. The PD weight function considers both geographic unit population information and the geographic distance of cell i and j. A higher weight is given to pairs with a shorter distance. The parameter λ in the PD function is chosen by the user and allows the user to view the population as a measure of spatial clustering, where large (small) λ is more sensitive to larger (smaller) clustering pattern. In this paper, we used λ as 50% of the total population, which allows for a large number of geographic neighbors for evaluation. For global clustering patterns (e.g. spiral or linear), many geographical units have a spatial correlation even if the distance between them is large and they are not adjacent. Therefore, the PD function with a large *λ *(i.e. which evaluates many geographic units) performs much better than the adjacent weight function.

Note that only different versions of Moran's *I *and I*_pop _were compared in this paper because the major purpose was to explore a way to improve Moran's *I*. We included I*_pop _for comparison because I*_pop _has not been studied by many (see Jackson et al. [11]), and it has been well known as an alternative method for Moran's *I *developed for data with heterogeneous populations. We did not include other methods, such as Tango's MEET [12], for global clustering evaluation in the comparison. Tango's MEET [12] has been shown to be the most powerful method for identifying global clustering patterns (see Jackson et al [11], Song and Kulldorff [21], and Huang et al. [10]). However, with the PD weight function, Moran's *I *and I*_pop _may perform as well as Tango's MEET. This issue will be explored further in future work.

Few spatial studies exist that explore data with homogenous populations. Spatial studies with homogenous populations allow for stronger power studies since confounding effects due to heterogeneous populations are removed [9]. For example, when performing a spatial study in California (with counties as the geographic unit), spatial statistics tend to detect clusters where there are two counties with large populations in close proximity (e.g., Los Angeles county and San Bernardino county). Counts or rates from areas with small populations are more unstable than those with large populations and they can be masked by areas with large populations[22,23]. Also, for most applied studies involving real data, researchers are more interested in the pattern of the response variable (e.g. disease rate) rather than the population pattern [15,24,25]. Therefore, collecting and analyzing data with well defined regions (homogeneously populated) will be very useful.

## Competing interests

The authors declare that they have no competing interests.

## Authors' contributions

MCJ and LH designed the study and prepared the manuscript. RCT conceived the study and developed the theory. QX developed the simulated data. MCJ, LH, and RCT interpreted the simulation results. All authors read and approved the final manuscript.

## Authors' information

MCJ is a Statistics Professor at American University. LH is a Mathematical Statistician at the Food and Drug Administration (FDA) in the Office of Biostatistics. QX is a Statistician at American University. RCT is the Associate Director in the Office of Biostatistics at the FDA. The work was conducted while LH was a contractor and RCT was a Program Director at the National Cancer Institute (NCI), NIH, Bethesda, MD. The views expressed by the authors are not necessarily of those of FDA or NCI.
